# Strain Effects on the Electronic and Optical Properties of Blue Phosphorene

**DOI:** 10.3389/fchem.2022.951870

**Published:** 2022-07-07

**Authors:** Lin Zhang, Zhen Cui

**Affiliations:** ^1^ School of Science, Xi’an University of Technology, Xi’an, China; ^2^ School of Automation and Information Engineering, Xi’an University of Technology, Xi’an, China

**Keywords:** strain effect, blue phosphorene, photocatalyst, compressive strain, tensile strain

## Abstract

Monolayer blue phosphorene (BlueP) systems were investigated under biaxial strain range from −10% to +10%. All these systems exhibit excellent stability, accompanying changes in the electronic and optical properties. BlueP becomes metallic at −10% strain and transforms into a direct semiconductor at 10% strain while maintaining indirect semiconductor behaviors at −8% to +8% strain. The bandgap of BlueP decreases linearly with strain, and tensile strain exhibits a more moderate bandgap modulation than compressive strain. The real part of the dielectric function of BlueP is enhanced under compressive strain, while the optical absorption in the visible and the infrared light regions increases significantly under tensile strain. The maximum absorption coefficient of 0.52 ×10^5^/cm occurs at 530 nm with the 10% strain. Our analysis indicates that the semiconductor–metal transition and the indirect–direct bandgap transition are the competition results of the energy states near the Fermi level under a massive strain. The potent compressive strain leads the *p*_*y*_ orbitals of the conduction band to move downward and pass through the Fermi level at the K point. The robust tensile strain guides the energy states at the Γ point to approach the Fermi level and become the band edges. Our results suggest that the energy storage capacity of BlueP can be significantly improved by compressive strain, while the visible light photocatalytic performance is enhanced by tensile strains of less than 8%. Our works provide a reference for the practical applications of BlueP in photocatalyst, photovoltaic cells, and electronic devices.

## Introduction

The miniaturization requirement of optoelectronic devices accelerated the exploration of multifunctional two-dimensional (2D) materials (Mélinon et al., 2007; Youngblood et al., 2015; Zhang and Cui, 2022a). 2D materials and structures, such as single-atom crystal (Du et al., 2016; Yu et al., 2016; Cui et al., 2021a), group-IV compounds (Peng et al., 2013; Sun et al., 2021; Zhang and Cui, 2022b), transition metal compounds (Cui et al., 2021b; Cui et al., 2021c), and van der Waals heterostructures (Memaran et al., 2015; Wang et al., 2018a), have been investigated by first-principles calculations. The results show that the flexible structure, adjustable bandgap, and excellent compatibility with traditional silicon-based devices make these 2D materials qualified not only as catalysts (Sun and Schwingenschlögl, 2020a; Luo et al., 2021; Liu et al., 2022), spintronics (Yuan et al., 2018; Li et al., 2021), nanomechanics (Sun and Schwingenschlögl, 2020b), energy conversion (Pospischil et al., 2014), and gas-sensing devices (Kooti et al., 2019; Sun et al., 2019), but also as optoelectronic devices (Sun et al., 2017a; Ghojavand et al., 2020; Liu et al., 2020; Cui et al., 2022).

As the allotrope of layered black phosphorus (BlackP) materials, blue phosphorene (BlueP) with a puckered honeycomb structure shares the high stability and carrier mobility with BlackP (Zhu and Tománek, 2014; Sun et al., 2019). The ∼2 eV bandgap makes BlueP more suitable than BlackP as a high-performance field effect transistor channel material (Liu et al., 2014). To achieve more applications, a series of bandgap modulation methods have been carried out by employing electric field (Ghosh et al., 2015), stacking effects (Mogulkoc et al., 2016; Pontes et al., 2018), doping (Sun et al., 2015; Zhang et al., 2017), functionalization (Zhu et al., 2016; Yang et al., 2017), and forming heterostructures with other materials (Sun et al., 2017b; Kaewmaraya et al., 2018; Mogulkoc et al., 2018). The absorption spectrum of BlueP-based devices has spanned from the ultraviolet to the infrared light (Sun et al., 2020). Recently, the convenience of synthesis and exfoliation (Gu et al., 2017; Zeng et al., 2017) further accelerates the practical applications in lithium-ion batteries (Bao et al., 2018; Li et al., 2018), photocatalysts (Wang et al., 2018b; Wang et al., 2018c; Ju et al., 2018; Gao et al., 2019), and gas sensors (Montes and Schwingenschlögl, 2017; Safari et al., 2018).

During the fabrication of monolayer nanostructures, the inevitable stress and strain influence the actual bandgap of 2D materials. However, it has been demonstrated as an effective and low-cost method to fulfill the continuous control of bandgap by employing elastic strain (Feng et al., 2012; Çakır et al., 2014; Peng et al., 2020; Kilic and Lee, 2021; Lou et al., 2021). To the best of our knowledge, the strain effects on the bandgap, electronic, and optical properties have seldom been discussed in BlueP. In this work, the properties of BlueP systems were studied under strain ranging from −10% to +10%. It is shown that the bandgap of BlueP decreases linearly with the strain. Compared with the compressive strain, the tensile strain exhibits a more moderate bandgap adjustment and strong absorption in the visible light region. BlueP shows the fascinating photocatalytic and photovoltaic properties under 8% tensile strains, while the energy storage capacity of BlueP is enormously improved under compressive strains. The semiconductor–metal transition and the indirect–direct bandgap transition occur at the −10% and the 10% strain, respectively. These transitions are attributed to the competition of the energy states nearby the Fermi level under a massive strain. Our works provide a theoretical reference for the actual applications of BlueP in photocatalyst, photovoltaics, and electronics.

## Computational Details

All our calculations are performed by the Vienna *ab initio* simulation package (Kresse and Furthmüller, 1996a). Generalized gradient approximation of the Perdew–Burke–Ernzerhof function analyzes the parameterized exchange–correlation interaction (Kresse and Furthmüller, 1996b; Kresse and Joubert, 1999). High computational accuracy is guaranteed by a 550-eV cut-off energy of plane-waves basis. An 11 × 11×1 Monkhorst–Pack k-point mesh is constructed. A vacuum layer of 20 Å height was employed to eliminate the influence of interlayers (Perdew et al., 1996). To ensure the system is tested at the steadiest state, the Hellmann–Feynman force on each atom and the total energy change are required to converge to 0.01 eV/Å and 10^−5^ eV/atom, respectively. Local field effects and frequency-dependent dielectric response theory are considered to obtain the optical properties in the random-phase approximation (RPA) (Hybertsen and Louie, 1986; Heyd et al., 2003; Grimme et al., 2010). The data processing is performed by VASPKIT (Wang et al., 2021).

## Results and Discussion

**FIGURE 1:**
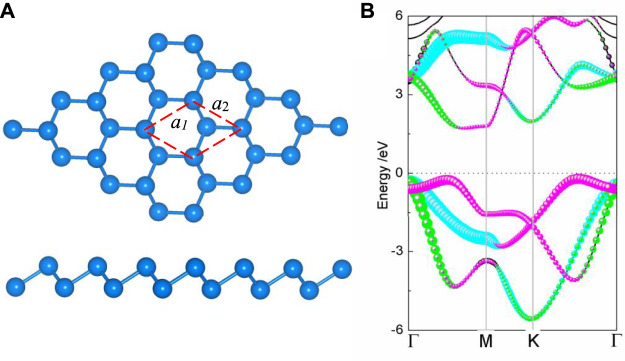
**(A)** Top and side views of crystal structure and **(B)** projected band structure of BlueP. The blue, green, and peach dot lines represent the energy levels contributed by the *p*_*x*_, *p*_*y*_, and *p*_*z*_ orbitals, respectively. The energy of the Fermi level is set to zero.

The puckered honeycomb structure of the relaxed monolayer BlueP is depicted in Figure 1A, where half of the atoms are squeezed out of the plane formed by the others. The vertical distance between the upper and lower atoms is 1.24 Å. Each BlueP is connected with three adjacent BlueP by covalence bands and forms a P–P bond angle of 92.7°. The lattice constants *a*_1_ and *a*_2_ are 3.20 Å, similar to the reported values (Ju et al., 2018; Sun et al., 2020). The indirect bandgap monolayer of BlueP is 1.87 eV. The conduction band minimum (CBM) is primarily contributed by the *p*_*z*_ and *p*_*y*_ orbitals, while the valence band maximum (VBM) by the *p*_*z*_ and *p*_*x*_ orbitals, as illustrated in Figure 1B.

The strain on BlueP is calculated by the value of (*a*–*a*_1_)/*a*_1_, where *a*_1_ and *a* are the lattice constants of the structures before and after strain, respectively. Tensile strain is positive, while compressive strain is negative. The physical and chemical properties of BlueP are investigated under the biaxial strains ranging from −10% to 10%. Our results show that BlueP presents excellent stability under strain.

**FIGURE 2:**
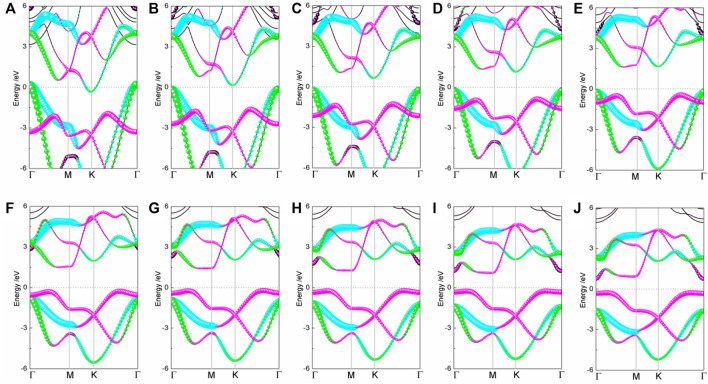
The projected band structures of BlueP under elastic strain **(A)** −10%, **(B)** −8%, **(C)** −6%, **(D)** −4%, **(E)** −2%, **(F)** 2%, **(G)** 4%, **(H)** 6%, **(I)** 8%, and **(J)** 10% (the blue, green, and peach dot lines denote the contributions of *p*_*x*_, *p*_*y*_, and *p*_*z*_ orbitals, respectively).

The energy structure of the strained BlueP exhibits a significant strain dependence, as shown in Figure 2. As the compressive strain increases, the *p*_*y*_ orbitals of the conduction band move downward and pass through the Fermi level at the K point under the −10% strain, causing the semiconductor–metal transition. Compared with the apparent movement of the *p*_*y*_ orbitals in the conduction band, compressive strain has fewer effects on the *p*_*y*_ and *p*_*x*_ orbitals in the valence band.

**FIGURE 3:**
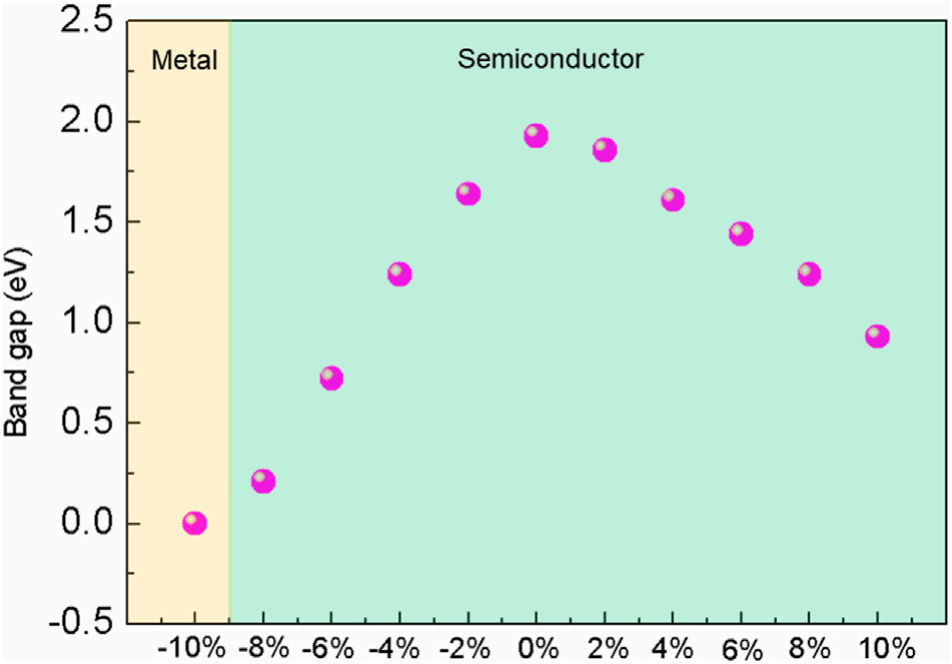
Relationship between the bandgap and the strain on monolayer BlueP.

On the tensile conditions, the energy states of *p*_*z*_ orbitals at the Γ point approach the Fermi level with strain and become the CBM and VBM gradually. At the 10% strain, BlueP experiences the indirect–direct semiconductor transition. The bandgap of BlueP decreases linearly with the strain for either tensile or compressive strain, as shown in Figure 3. Compared with the compressive strain, the tensile strain makes a more moderate bandgap modulation, which is crucial for the accurate bandgap control.

The absorption spectrum from the ultraviolet to visible light is essential in optical and renewable energy systems. Dielectric function is introduced to investigate the electrical and optical properties of BlueP (Toll, 1956; Ehrenreich and Cohen, 1959)

ε=ε(ω)1+iε2(ω), (1)

where *ω* represents the angular frequency of the electromagnetic wave, and complex dielectric function *ε* reflects the electric polarizability capacity of the material. *ε*_1_(*ω*) and *ε*_2_(*ω*) are the real part and the imaginary part of the *ε*, respectively. *ε*_1_(*ω*) describes the ability to store energy, while the *ε*_2_(*ω*) represents the loss.

The absorption coefficient is calculated by the following expression:

α(ω)=2ω[ε12(ω)+ε22(ω)−ε(ω)12]1/2. (2)

**FIGURE 4:**
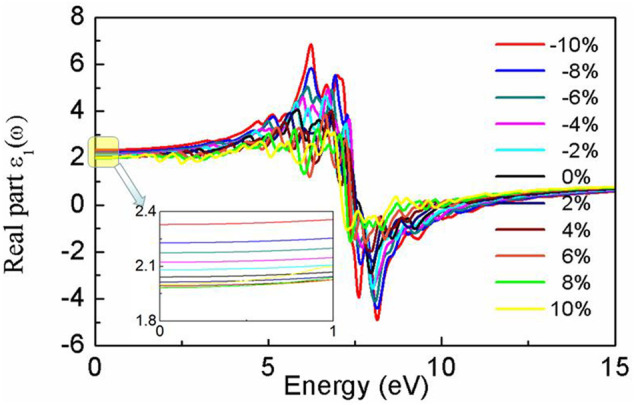
The real part of the dielectric function of BlueP with different strains.

Our results show that the dielectric constant *ε*_1_ is highly dependent on strain, as depicted in Figure 4. *ε*_1_ (0) is 2.04 for the relaxed BlueP; it varies from 1.98 to 2.32 for the strained BlueP. Compressive strain exhibits more influence on ε_1_ (0) than the tensile strain. On the one hand, *ε*_1_ (0) decreases with the increase of tensile strain, saturating to 1.98 under the 6% strain. On the other hand, *ε*_1_ (0) exhibits an increasing tendency with compressive strain, achieving the maximum of 2.32 at −10% strain.

The peak of *ε*_1_(ω) decreases with the tensile strain, accompanying a gentle red-shift. However, the peak increases with compressive strains and blueshifts. The larger the compressive strain is, the higher the peak of *ε*_1_(ω), and the greater the blueshift. At the −10% strain, *ε*_1_(*ω*) has a positive maximum of 7.0 at 5.84 eV and a negative maximum of −4.0 at 8.22 eV. This dramatic increment of *ε*_1_(*ω*) indicates that compressive strain can enhance the energy storage capacity of BlueP.

**FIGURE 5:**
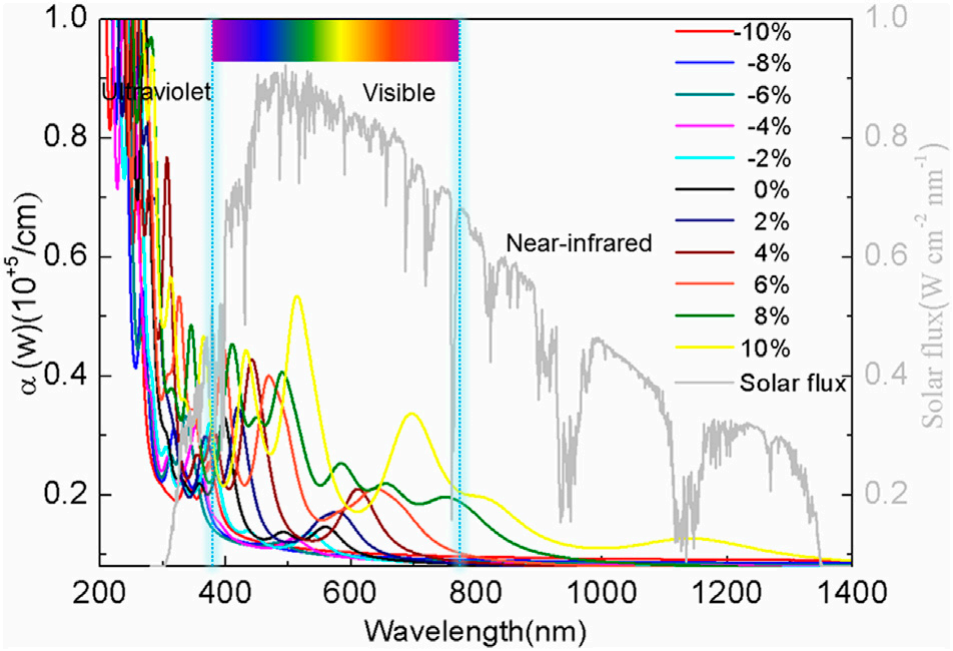
The optical absorption spectra of BlueP under different strains.

In addition, the absorption spectra of BlueP were compared with the solar spectrum (NREL), as shown in Figure 5. It is shown that the absorption spectrum of the relaxed BlueP spans from the ultraviolet to the green-light region. The absorption of the ultraviolet light is relatively strong, while it is weak in the visible light region, which indicates that the relaxed BlueP is not an excellent visible light photovoltaic material (Sun et al., 2020). The absorption spectrum of BlueP blueshifts under compressive strains, while the absorption of the visible light decreases. It suggests that compressive strain does not improve the absorption ability of BlueP.

On the tensile strain side, the absorption spectrum of BlueP redshifts, accompanying a significant increase of absorption of the visible and the infrared lights. The larger the tensile strain is, the greater the peak of the absorption coefficient, and the greater the red-shift are. At the 10% strain, the absorption coefficient achieves the maximum of 0.52 ×10^5^/cm at 530 nm wavelength. BlueP presents preeminent optical properties, which is critical in the photocatalyst and photovoltaic cells.

The photocatalytic performance of BlueP was further discussed. As we know, excellent water-splitting photocatalysts put forward requirements for the bandgap and the band edges of materials (Bao et al., 2018; Gao et al., 2019; Kilic and Lee, 2021): a bandgap larger than the energy requirement for water splitting, a CBM higher than the reduction potential energy (H^+^/H_2_), and a VBM lower than the oxidation potential energy (O_2_/H_2_O). Additionally, a higher absorption of the solar spectrum is also crucial.

**FIGURE 6:**
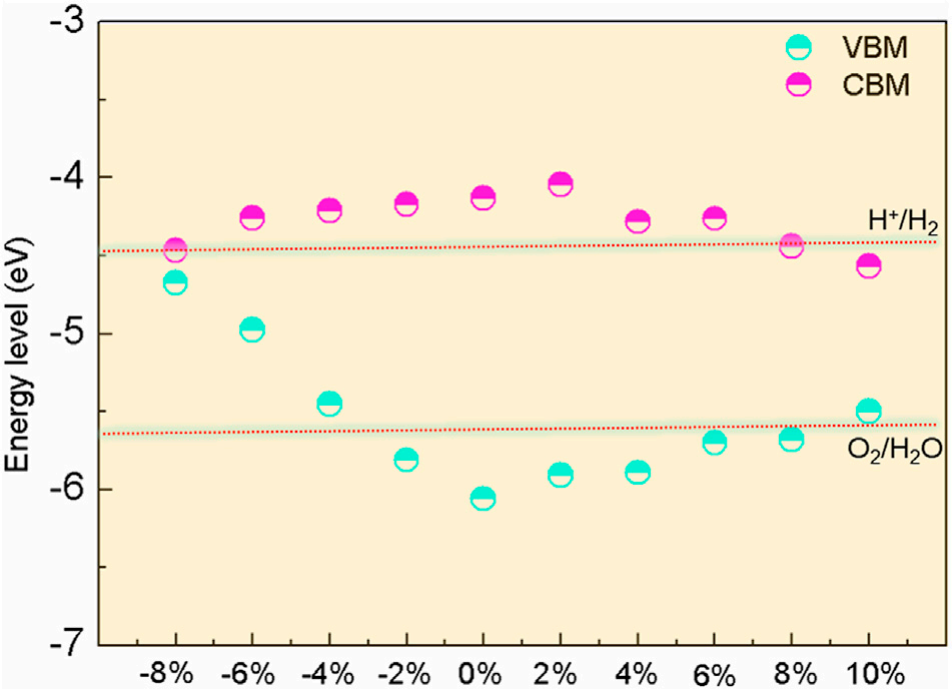
The band edge positions of BlueP with different strains against the redox potential of the water splitting.

The band edge positions of BlueP concerning the vacuum level are illustrated in Figure 6. The potentials of the reduction and the oxidation at the acidic environment pH = 0 are marked. It can be seen that the bandgap of BlueP satisfies the water-splitting reaction requirement under strain from −4% to 8%. The reduction (H^+^/H_2_) can be improved under the strain range of −8% to 8%, while the oxidation (O_2_/H_2_O) promoted under the strains between −2% and 8%. Considering the weaker optical absorption under compressive strain, BlueP exhibits an excellent photocatalytic performance under the tensile strains of less than 8%. What should be pointed out is that BlueP is a single-atom material. The photocatalytic property of BlueP will be weakened by the increasing recombination rate of electrons and holes because the accumulation of electrons and holes is on the same BlueP surface (Qu and Duan, 2013; Ju et al., 2018; Ren et al., 2020). However, this problem can be overcome by forming BlueP-based heterostructures with other materials, where electrons and holes accumulate on different material surfaces (Dheivanayagam et al., 2016; Zhou et al., 2016; Zhang et al., 2021).

## Conclusion

In this work, the optical and electronic properties of monolayer BlueP systems under biaxial strain were investigated. All systems exhibit excellent stability under the biaxial strain of −10% to +10%. The bandgap of BlueP decreases with strain, while the tensile strain makes a more moderate bandgap modulation. BlueP remains the behavior of indirect semiconductor under the strains of −8% to +8%, while a metal changes at the −10% strain and becomes a direct semiconductor at the 10% strain. Although exhibiting a weaker absorption in the relaxed state, the visible light absorption of BlueP significantly increases under tensile strain, accompanying an apparent red-shift. The larger the tensile strain is, the greater the peak of the absorption coefficient, and the larger the red-shift is. The absorption coefficient has a maximum of 0.52 ×10^5^/cm at 530 nm under the 10% strain. BlueP exhibits an excellent photocatalytic property under the tensile strain of less than 8%. The increasing *ε*_1_ indicates that the energy storage capacity of BlueP can be enhanced by compressive strain. Our analysis indicates that the semiconductor–metal transition and the indirect–direct bandgap transition are the competition results of the energy states nearby the Fermi level under massive strains. The intense compressive strain causes the significant decrease of the *p*_*y*_ orbitals of the CBM at the K points, while the potent tensile strain guides the energy states at the Γ point to approach the Fermi level and becomes the band edges gradually. This study provides references for BlueP applications in photocatalyst, photovoltaics, and electronics.

## Data Availability

The original contributions presented in the study are included in the article/Supplementary Material; further inquiries can be directed to the corresponding author.
